# Tinnitus Among Patients With Anxiety Disorder: A Nationwide Longitudinal Study

**DOI:** 10.3389/fpsyt.2020.00606

**Published:** 2020-06-25

**Authors:** Sheue-Jane Hou, Albert C. Yang, Shih-Jen Tsai, Cheng-Che Shen, Tsuo-Hung Lan

**Affiliations:** ^1^ Institute of Clinical Medicine, National Yang-Ming University, Taipei, Taiwan; ^2^ Department of Psychiatry, Cheng Hsin General Hospital, Taipei, Taiwan; ^3^ Institute of Brain Science, National Yang-Ming University, Taipei, Taiwan; ^4^ Division of Interdisciplinary Medicine and Biotechnology, Beth Israel Deaconess Medical Center, Harvard Medical School, Boston, MA, United States; ^5^ Department of Psychiatry, Taipei Veterans General Hospital, Taipei, Taiwan; ^6^ Division of Psychiatry, National Yang-Ming University, Taipei, Taiwan; ^7^ Department of Psychiatry, Chiayi Branch, Taichung Veterans General Hospital, Chiayi, Taiwan; ^8^ Tsaotun Psychiatric Center, Ministry of Health and Welfare, Nantou, Taiwan

**Keywords:** anxiety disorder, tinnitus, hazard ratio, National Health Insurance research database, cohort study

## Abstract

**Objectives:**

The association between tinnitus and anxiety disorder remains debated. We used a retrospective cohort study to investigate the relationship between anxiety disorder and tinnitus, aiming to decipher possible risk factors for tinnitus in patients with anxiety disorder.

**Method:**

Data on a total of 7,525 patients with anxiety disorder and 15,050 patients without (comparison cohort) were extracted from the Longitudinal Health Insurance Database 2005 in Taiwan. The Kaplan–Meier estimator with the log rank test and the Cox proportional-hazard regression model were used to compare the incidence of tinnitus in both groups and to identify risk factors that predicted tinnitus.

**Results:**

After adjusting for related covariates, the hazard ratio for the development of tinnitus during the follow-up period was 3.54 (95% confidence interval: 3.11–4.02, *P* < .001) for anxiety disorder cohort relative to comparison cohort. Age ≧ 60 years, female sex, hypertension, and hyperlipidemia were statistically significant predictive risk factors of tinnitus in patients with anxiety disorder.

**Conclusion:**

A significant increase in the lifetime incidence of tinnitus was exhibited in patients with anxiety disorder. Elderly subjects, female sex, hypertension, and hyperlipidemia were risk factors. Clinicians should be alert to the possibility of tinnitus in subjects with anxiety disorder.

## Introduction

Tinnitus, the perception of an internal sound without an external sound source, is a common audiological symptom globally. Its prevalence has been reported to be 20.7% in Korea (1), 18.6% in Japan (2), 14.5% in China (3), 25.3% in the US (4), and 18.4% in England (5). In the Korean population, the prevalence of tinnitus increased sharply after 55 years of age and peaked at 70 (1). In south Taiwan, Chang *et al* reported a 32% prevalence of persistent tinnitus among people older than 65 years (6).

Several studies have indicated strong correlation between anxiety and tinnitus. However, the causal relationship remains undetermined. Salviati et al. evaluated 156 patients with chronic tinnitus using the Tinnitus Handicap Inventory, the Symptomatic Check List-90-Revised, and the Stress-Related Vulnerability Scale, finding that 43.59% were affected by a psychiatric disorder (7). In addition to having fewer mean total sleep hours per night, a greater value for the mean number of work days missed, and higher rates of depressive disorders, 26.1% of 21 million individuals with tinnitus in the United States reported experiencing anxiety problems in the preceding 12 months, whereas only 9.2% of people who had no tinnitus experienced anxiety problems (8). Higher rates of all anxiety disorders were also observed in several European studies’ participants with tinnitus relative to the general population (9–11). In Taiwan, an increased prevalence and risk of anxiety disorders in adults with tinnitus were also reported by Lin et al. (12). Relative to those without tinnitus, patients with severe tinnitus were at increased risk of anxiety symptoms (odds ratio [OR] = 1.11) in a large middle-aged UK population (13). Kehrle et al. studied 84 individuals with tinnitus and normal hearing levels in Brazil and noted a positive correlation between tinnitus annoyance and anxiety severity (14). These study results suggest that the perceived severity of tinnitus is influenced by comorbid anxiety disorder (15).

A possible relationship between anxiety disorder and tinnitus has been strongly suggested by these studies. However, from an epidemiological viewpoint, correlation does not imply causation. Pattyn et al., in their review of the literature on tinnitus and anxiety disorders, stated that “*We found no studies on the prevalence of tinnitus in populations with anxiety disorders. As only one direction of the relationship is known, this precludes any conclusion as to the opposite direction. We also found no longitudinal studies on this subject and hence have cross-sectional, correlation-only results.*” (16). Salviati et al. (17) and Zoger et al. (18) have attempted to clarify the onset sequence of tinnitus and anxiety disorder, but recall bias could have been present.

One aim of this study was to use the Longitudinal Health Insurance Database 2005 (LHID 2005) to examine the relationship between anxiety disorders and tinnitus. A second goal was to identify risk factors for patients with anxiety disorder who develop tinnitus.

## Material and Methods

### Data Sources

Instituted in 1995, the National Health Insurance (NHI) program is a mandatory health insurance program that offers comprehensive medical care coverage, including outpatient, inpatient, and emergency care and traditional Chinese medicine, to all residents of Taiwan; the coverage rate is as high as 99% (19). The NHI research database (NHIRD) contains comprehensive information regarding clinical visits, including prescription details and diagnostic codes based on the International Classification of Diseases, Ninth Revision, Clinical Modification (ICD-9-CM). The NHIRD is managed by the National Health Research Institute (NHRI), and confidentiality is maintained in accordance with directives of the Bureau of the NHI. The data source for our study was the LHID 2005, which is a dataset in the NHIRD. Data for the LHID 2005 were collected systematically and randomly sampled from the NHIRD; the database included data from one million individuals. There were no significant differences in sex distribution, age distribution, or average payroll-related insurance costs between the patients in the LHID and those in the original NHIRD (20).

### Ethics Statement

The Institutional Review Board of the Taipei Veterans General Hospital approved this study (VGHIRB No.: 2018-07-016 AC). Written consent from study participants was not obtained because the NHI dataset comprises de-identified secondary data that can be used for research and the Institutional Review Board of Taipei Veterans General Hospital issued a formal written waiver for the requirement of consent.

### Study Population

Using data extracted from the LHID, we conducted a retrospective cohort study of patients aged 20 years and older who were newly diagnosed with anxiety disorder between January 1, 2000 and December 31, 2004. Anxiety disorder was defined according to the ICD-9-CM codes 300.0, 300.2, 300.3, 308.3, and 309.8 in Ambulatory care expenditures by visits (CD) and Inpatient expenditures by admissions (DD) files of LHID 2005. To ensure diagnostic validity and patient homogeneity, we included only patients diagnosed by psychiatrists (FUNC_TYPE = 13) with at least two consistent anxiety diagnoses to improve diagnostic validity. Division of visit was obtained according to FUNC_TYPE in CD and DD files of LHID 2005. We excluded patients who were diagnosed with anxiety disorder between January 1, 1996 and December 31, 1999. We also excluded patients who were diagnosed with tinnitus (ICD-9-CM code 388.3) before they were diagnosed with anxiety disorder. For each patient included in the final cohort, two age- and sex-matched comparison participants who had not been diagnosed with anxiety disorder or tinnitus were randomly selected from the LHID 2005. All participants in the anxiety disorder cohort and comparison cohort were observed until diagnosed with tinnitus by an otolaryngologist (FUNC_TYPE=09) or a neurologist (FUNC_TYPE = 12) or until December 31, 2009. The primary clinical outcome was otolaryngologist- or neurologist-diagnosed tinnitus. Insurance premiums, which were calculated according to the beneficiary’s total income, were used to estimate monthly income. Monthly income was stratified into low income (<NT$ 20,000), medium income (NT$ 20,000–NT$ 39,999), and high income (≧NT$ 40,000). Urbanization level was stratified into urban, suburban, and rural. All communities in Taiwan were stratified into seven levels based on a composite score obtained by calculating the population density, the proportion of people in the population with a college or above educational level, the proportion of people over the age of 65, the proportion of the population engaged as agricultural workers, as well as the number of physicians per 100,000 people (21). In our study, we further categorized seven levels of urbanization into urban (levels 1–2), suburban (levels 3–4), and rural (levels 5–7). Urbanization and monthly income levels were used to represent socioeconomic status. For sensitivity analyses, we also conducted a cohort of newly diagnosed anxiety disorder patients who were diagnosed by psychiatrists or nonpsychiatrists between January 1, 2000 and December 31, 2004.

### Statistical Analyses

Generalized estimating equations under an independent structure of working correlation matrix were used to examine the differences in the demographic characteristics between the participants in anxiety disorder cohort and comparison cohort. The incidence of newly diagnosed tinnitus in the anxiety disorder cohort and comparison cohort was calculated, and the rate ratio (RR) between two cohorts was also calculated. To investigate potential surveillance bias, subgroups were stratified according to the duration since diagnosis of anxiety disorder. Subgroups were also stratified according to age and sex. Survival analyses of censored data were constructed using the Kaplan–Meier estimator. The log-rank test was used to compare survival data curves. 

Cox proportional-hazard regression was used to identify risk factors that predicted tinnitus. Variables, such as anxiety disorder, age, sex, common comorbidities (hypertension, diabetes mellitus, dyslipidemia, coronary artery disease, congestive heart failure, chronic lung disease, malignant neoplasms, head injury, and cerebrovascular disease), urbanization, and monthly income were included as covariates in the univariate model. Factors that demonstrated a moderately significant statistical relationship in the univariate analysis (*P* < .1) were entered by forward selection in a multivariable Cox proportional-hazard regression model. Univariate and multivariable Cox models were also used for a subgroup analysis in patients with anxiety disorder to identify risk factors that predicted tinnitus in patients with anxiety disorder. The level of statistical significance was established at *P *< .05. Furthermore, we also tested the assumption of proportional hazard in our work by using log-minus-log survival plot.

The Perl programming language (version 5.12.2) was used to extract and compute data. Microsoft SQL Server 2005 (Microsoft Corp., Redmond, WA, USA) and SAS statistical software (version 9.2; SAS Institute Inc., NC, USA) were used to perform all statistical analyses.

## Results

**Figure 1:**
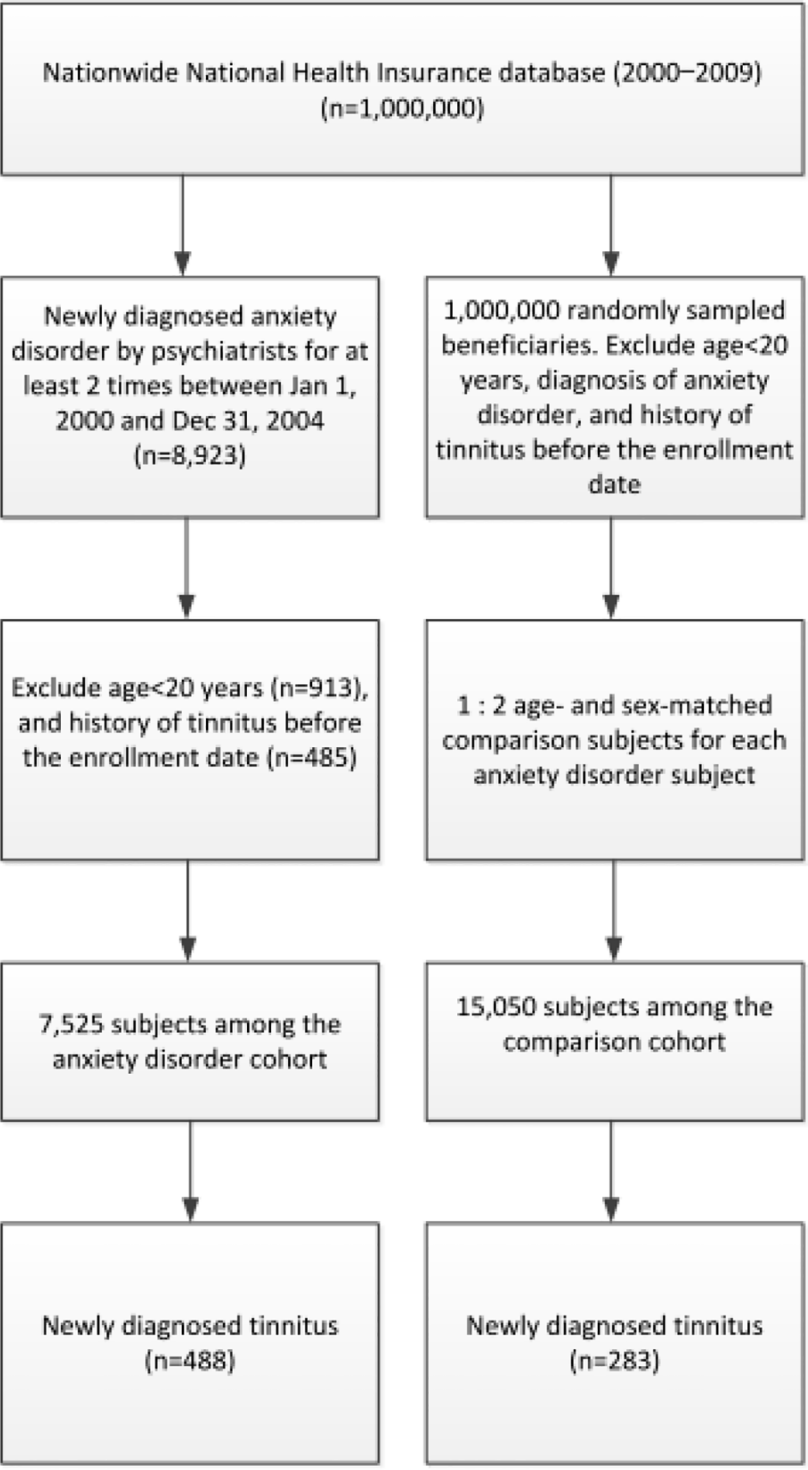
Flowchart of participants selection. NHIRD, National Health Insurance Research Database.

Our study included 7,525 subjects with anxiety disorder and 15,050 matched comparison participants), of whom 60.3% were women (**Figure 1**). The median age at enrollment was 42 years (interquartile range [IQR]: 32–53 years), and the median follow-up periods in the anxiety cohort and comparison cohort were 7.3 (IQR: 5.9–8.7) and 7.5 years (IQR: 6.1–8.8 years), respectively. During the follow-up period, 488 (6.5%) subjects in the anxiety disorder cohort and 283 (1.9%) subjects in the comparison cohort were diagnosed with tinnitus (*P* <.001). The comorbidities analyzed, namely hypertension, diabetes mellitus, dyslipidemia, coronary artery disease, congestive heart failure, chronic lung disease, malignancy, head injury, and cerebrovascular disease, were more frequent in the anxiety disorder cohort than in the comparison cohort (**Table 1**).

**Table 1:** Baseline Characteristics of Patients With and Without Anxiety Disorder.

Demographic data				Patients with Anxiety disorder *N* = 7,525		Patients without Anxiety disorder *N* = 15,050		*P* value
				*n*	%	*N*	%	
Age (years)^a^				42 (32–53)		42 (32–53)		>.999
	≥60			1,182	15.7	2,376	15.8	
	<60			6,343	84.3	12,674	84.2	
Sex								>.999
	Male			2,991	39.7	5,982	39.7	
Female			4,534	60.3	9,068	60.3	
Comorbidities								
	Hypertension			1,607	21.4	1,987	13.2	<.001*
	Diabetes mellitus			833	11.1	1,143	7.6	<.001*
	Dyslipidemia			1,186	15.8	1,371	9.1	<.001*
	Coronary artery disease			68	0.9	65	0.4	<.001*
	Congestive heart failure			160	2.1	191	1.3	<.001*
	Chronic lung disease			1,012	13.4	1,133	7.5	<.001*
	Malignant neoplasms			99	1.3	143	1.0	.012*
	Head injury			1,394	18.5	1,593	10.6	<.001*
	Cerebrovascular disease			361	4.8	369	2.5	<.001*
Degree of urbanization								.001*
		Urban		4,893	65.0	9,467	62.9	
		Suburban		2,109	28.0	4,407	29.3	
		Rural		523	7.0	1176	7.8	
Income group								.005*
			Low income	3,597	47.8	6,885	45.7	
			Medium income	2,818	37.4	5,827	38.7	
			High income	1,110	14.8	2,338	15.5	
Newly diagnosed tinnitus				488	6.5	283	1.9	<.001*
Follow-up years^a^				7.3 (5.9–8.7)		7.5 (6.1–8.8)		<.001*

^a^Median (interquartile range); *statistical significance.

**Figure 2:**
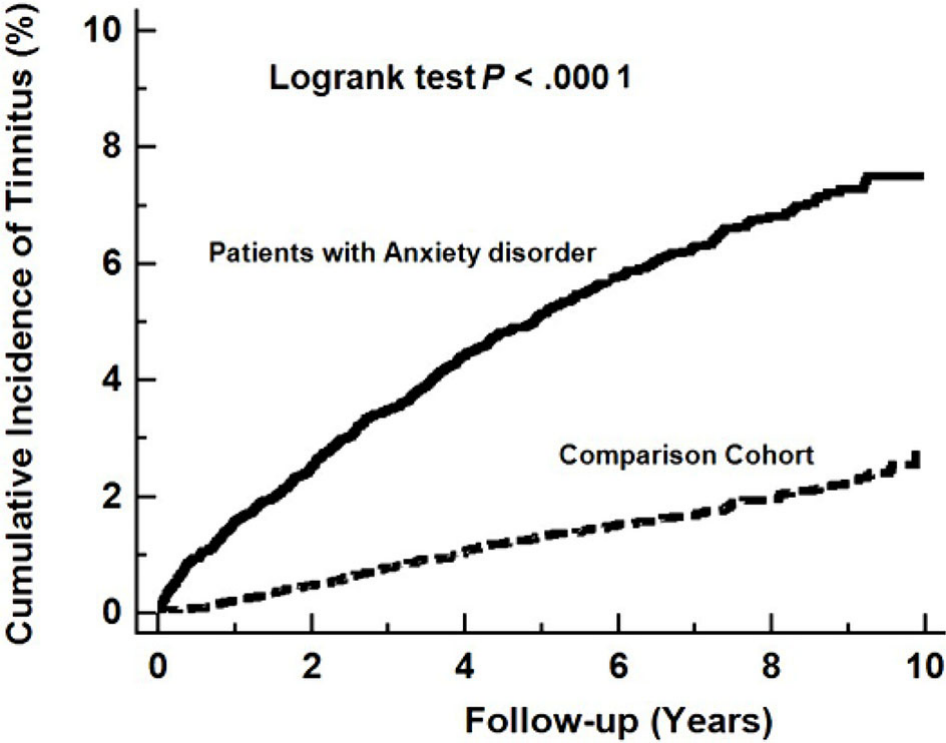
**Kaplan**–**Meier Curve**, demonstrating significantly higher cumulative incidence of tinnitus among patients with anxiety disorder *versus* the controls (log-rank test, *P* <.0001).

**Table 2** details the incidence of tinnitus in the anxiety disorder cohort and in the comparison cohort during the follow-up period. The average annual incidence of tinnitus was higher in the anxiety disorder cohort than in the comparison cohort (RR: 3.57, 95% confidence interval [CI]: 3.08–4.14, *P* <.001]. Compared with the comparison cohort, tinnitus was more common in the anxiety disorder cohort in both sex groups (RR of men: 4.15, 95% CI: 3.29–5.25, *P* <.001; RR of women: 3.19, 95% CI: 2.62–3.89) and in both age groups older or younger than 60 years (RR for age ≧ 60 years group: 3.46, 95% CI, 2.60–4.61, *P* <.001; RR for age < 60 years group: 3.63, 95% CI: 3.05–4.34, *P* <.001). With respect to the length of follow-up, the RR of tinnitus in the anxiety disorder cohort decreased from 7.63 (95% CI: 5.12–11.66) in the first year to 3.38 (95% CI: 2.77–4.13) after 1–5 years and then to 2.44 (95% CI: 5.12–11.66) after 5 years. The Kaplan–Meier survival analysis with a log-rank test revealed a significant association between anxiety disorder and tinnitus incidence (*P* <.0001) (**Figure 2**).

**Table 2:** Incidence of tinnitus in patients with and without anxiety disorder.

		Patients with Anxiety disorder		Patients without Anxiety disorder		Rate ratio (95% CI)	*P* value
		No. of Tinnitus	Per 1000 person–years	No. of Tinnitus	Per 1000 person-years				
Total		488	9.04	283	2.53	3.57 (3.08–4.14)	<.001
Age							
≥60		133	16.26	82	4.71	3.46 (2.60–4.61)	<.001
<60		355	7.75	201	2.13	3.63 (3.05–4.34)	<.001
Sex							
Male		223	10.52	112	2.54	4.15 (3.29–5.25)	<.001
Female		265	8.08	171	2.53	3.19 (2.62–3.89)	<.001
Follow-up							
<1		118	2.19	32	0.29	7.63 (5.12–11.66)	<.001
1≤ duration<5		266	4.93	163	1.46	3.38 (2.77–4.13)	<.001
≥5		104	1.93	88	0.79	2.44 (1.82–3.29)	<.001

CI, confidence interval.

After adjustment for age, sex, comorbidities, urbanization, and monthly income, the hazard ratio (HR) for the development of tinnitus disorder during the follow-up period was 3.54 (95% CI: 3.11–4.02, *P* <.001) in the anxiety disorder cohort relative to the comparison cohort (**Table 3**).

**Table 3:** Risk factors for tinnitus in patients with and without anxiety disorder.

Predictive variables		Univariate analysis			Multivariable analysis		
		HR (95% CI)	*P* value		HR (95% CI)	*P* value	
Anxiety		3.64 (3.23–4.11)	<.001		3.54 (3.11–4.02)	<.001*	
Age (<60 = 0, ≥60 = 1)		0.50 (0.11–2.36)	.381				
Sex (Female = 1, Male = 0)		0.85 (0.74–0.98)	.025		0.88 (0.76–1.02)	.082	
Comorbidities						
Hypertension		1.69 (1.41–2.02)	<.001		1.00 (0.80–1.25)	.969	
Diabetes mellitus		1.55 (1.26–1.89)	<.001		1.20 (0.93–1.56)	.163	
Dyslipidemia		1.91 (1.57–2.32)	<.001		1.35 (1.05–1.73)	.021*	
Coronary artery disease		0.80 (0.31–2.10)	.651				
Congestive heart failure		1.51 (0.95–2.40)	.084		0.87 (0.50–1.51)	.613	
Chronic lung disease		1.62 (1.32–1.99)	<.001		1.16 (0.89–1.51)	.288	
Malignant neoplasms		0.73 (0.37–1.47)	.383				
Head injury		1.27 (1.04–1.55)	.017		0.92 (0.70–1.20)	.523	
Cerebrovascular disease		1.94 (1.40–2.69)	<.001		1.45 (0.96–2.18)	.074	
Degree of urbanization							
Urban		Reference					
Suburban		1.08 (0.92–1.27)	.350		1.05 (0.88–1.26)	.591	
Rural		0.79 (0.61–1.04)	.097		0.76 (0.56–1.02)	.073	
Income group							
Low income		Reference					
Medium income		1.04(0.83–1.32)	.567				
High income		1.05 (0.89–1.23)	.720				

HR, hazard ratio; CI, confidence interval. *statistical significance.

In sensitivity analyses, 62,036 anxiety disorder patients who were diagnosed by psychiatrists or nonpsychiatrists and 62,036 subjects without anxiety disorder were included. Compared with the subjects in the comparison cohort, those in the anxiety disorder cohort exhibited a significantly higher risk of a subsequent tinnitus (HR = 1.80, 95% CI =1.72–1.88, *p* <0.001) (**Supplementary Table 1**).

Furthermore, univariate and multivariable Cox proportional-hazard regression analyses were performed to identify variables that predicted tinnitus in patients with anxiety disorder. The results indicated that age ≧ 60 years (HR: 1.52, 95% CI: 1.20–1.94, *P* <.001), female sex (HR: 1.23, 95% CI: 1.03–1.47, *P* = .026), and comorbidities including hypertension (HR: 1.37, 95% CI: 1.09–1.71, *P* = .007), and dyslipidemia (HR: 1.27, 95% CI: 1.00–1.61, *P* = .049) are significant risk factors for tinnitus in patients with anxiety disorder (**Table 4**).

**Table 4:** Risk factors for tinnitus in patients with anxiety disorder.

Predictive variables		Univariate analysis			Multivariable analysis		
		HR (95% CI)	*P* value		HR (95% CI)	*P* value	
Age (<60 = 0, ≥60 = 1)		2.09 (1.71–2.56)	<.001		1.52 (1.20–1.94)	.001*	
Sex (Female = 1, Male = 0)		1.30 (1.08–1.55)	.004		1.23 (1.03–1.47)	.026*	
Comorbidities						
	Hypertension	1.90 (1.57–2.30)	<.001		1.37 (1.09–1.71)	.007*	
	Diabetes mellitus	1.79 (1.41–2.26)	<.001		1.23 (0.95–1.60)	.123	
	Dyslipidemia	1.72 (1.40–2.12)	<.001		1.27 (1.00–1.61)	.049*	
	Coronary artery disease	0.70 (0.22–2.17)	.535				
	Congestive heart failure	1.65 (1.00–2.71)	.050		0.95 (0.57–1.60)	.856	
	Chronic lung disease	1.47 (1.17–1.85)	.001		1.13 (0.88–1.44)	.343	
	Malignant neoplasms	0.31 (0.08–1.25)	.101				
	Head injury	0.94 (0.75–1.17)	.602				
	Cerebrovascular disease	1.60 (1.13–2.25)	.008		1.04 (0.73–1.49)	.832	
Degree of urbanization							
	Urban	Reference					
	Suburban	1.26 (1.04–1.52)	.021		1.22 (1.00–1.48)	.047*	
	Rural	1.22 (0.87–1.71)	.253		1.06 (0.75–1.50)	.742	
Income group							
	Low income	Reference					
	Medium income	0.86 (0.71–1.05)	.136		0.97 (0.79–1.18)	.724	
	High income	0.75 (0.57–1.00)	.049		0.90 (0.67–1.21)	.500	

The log-minus-log survival plot showed approximately two parallel lines in the cumulative hazard rate during the follow-up period. The result showed that there is no evidence that the proportional-hazards assumption has been violated in the cox regression model in our study (**Supplementary Figure 1**).

## Discussion

Many studies have revealed close correlation between tinnitus and anxiety disorders through cross-sectional analysis. To our knowledge, this is the first longitudinal study to compare the incidence of tinnitus in patients with and without anxiety disorder. Through an analysis of Kaplan–Meier curves, a statistically significant relationship was found between anxiety disorder and tinnitus. Relative to the comparison cohort, the incidence of tinnitus in the anxiety disorder cohort was much higher (9.04 *vs.* 2.53 per 1,000 person-years), and the incidence RR was 3.57 (95% CI: 3.08–4.14). There are several potential explanations for these findings. The first is that anxiety disorder causes tinnitus, the second is that anxiety disorder and tinnitus exhibit a co-causality relationship, and the third is that some common factors, such as genetic factors or dysfunction of central nervous system networks, result in both diseases. Because the possible roles of these unobserved missing variables are unknown, causal inference was tenuous (22). Many researchers have studied brain networks (23), central nervous structures (24–26), or hearing pathways (27) to link anxiety disorder and tinnitus. The limbic system, including the amygdala and hippocampus, the locus coeruleus (LC), the hypothalamus (28), the hypothalamic–pituitary–adrenal axis (HPA) (29), and the dorsal cochlear nucleus (DCN) (27) have been postulated to be important parts that are involved in tinnitus and anxiety arousal.

The amygdala, receives sensory information from the thalamus and the prefrontal cortex et al, processes anxiety, and regulates multiple regions’ reactions to aversive stimuli (30). The hippocampus is involved in memory processing, learning, and emotions (31). The LC is located in the upper dorsolateral pontine tegmentum; contains norepinephrine-synthesizing neurons; and plays a major role in arousal, attention, and stress responses (32). The auditory system is transported by the inner ear auditory nerve to the DCN and reaches to the primary auditory cortex and associated auditory areas of the brain (33). The HPA axis is the main neuroendocrine signaling system, which involves physiological homeostasis and stress responses (34). Genetic factors, alterations in the limbic system and hyperactivity of the HPA axis are considered to be the fundamental biological mechanisms that underlie anxiety disorders (35). HPA-induced stress hormones, corticosteroids (glucocorticoid and mineralocorticoid) are released and act through their respective receptors to induce genomic and nongenomic changes to the auditory system. Both acute and chronic stresses have been demonstrated to influence glutamate neurotransmission and thereby contribute to neuronal plasticity (36, 37). Mazurek et al. postulated that stress may induce neuronal plasticity, causing auditory pathologies and tinnitus, but this remains to be verified (38). In addition, the DCN exhibits direct connections with non-auditory brainstem structures, the LC, reticular formation, and raphe nuclei, which are involved in emotional control. Locus coeruleus hyperactivity could influence fusiform cells in the DCN and contribute to the onset and modulation of tinnitus (27). Through the aforementioned possible mechanisms, anxiety disorder is a probable cause of tinnitus.

A cross-sectional study using the NHIRD in Taiwan found that the prevalence of anxiety disorder in tinnitus and general population groups was 3.9 and 1.5% (12). They revealed that patients with tinnitus were significantly associated with increased risk of ADs (adjusted odds ratio = 1.99; 95% CI = 1.81–2.19; P < 0.001), and patients with anxiety disorder had an increased risk of tinnitus (adjusted odds ratio = 2.04; 95% CI=1.86–2.25; P < 0.001) (12). The design of aforementioned study prevented the temporal causal relationship between anxiety disorder and tinnitus from being clarified. Furthermore, it has not been possible to observe the incidence and sequential risk of tinnitus among anxiety disorder patients from such cross-sectional study. Therefore, we conducted this cohort study.

In the present study, we conducted a subgroup analysis where data were stratified by the time from the diagnosis of anxiety disorder to the new onset of tinnitus (**Table 2**). The results indicated that incident tinnitus increased not only within the first year but also after the first and the fifth years of an anxiety disorder diagnosis. Generally, subjects with anxiety disorder are more likely to undertake more frequent outpatient visits relative to the general population, leading to an earlier diagnosis of tinnitus and causing surveillance bias. However, our result suggests that the increased risk of tinnitus in subjects with anxiety disorder in this study cannot be wholly attributable to surveillance bias.

In subjects with anxiety disorder, being of an age greater or equal to 60 years was positively associated with having tinnitus (HR: 1.52, 95% CI: 1.20–1.94, *P = .*001), a finding consistent with other studies (1, 39). In addition to age, our results revealed a statistically significant association between tinnitus and hypertension (HR: 1.37, 95% CI: 1.09–1.71, *P* = .007) and between tinnitus and dyslipidemia (HR: 1.27, 95% CI: 1.00–1.61, *P* = .049). These findings were consistent with the study by Martines et al. (40). The potential underlying mechanisms may include high blood pressure causing damage to the cochlear microcirculation, diverse antihypertensive drugs leading to ototoxicity, and hyperlipidemia affecting part of the cochlea (41). Thus, these major cardiovascular risk factors may have adversely affected tinnitus, and more studies are required for confirmation.

Female study participants constituted 60% of our study sample. Female sex was associated with an increased risk of tinnitus in subjects with anxiety (HR: 1.23, 95% CI: 1.03–1.47, *P* = .026). Bhatt’s cross-sectional study in 2018 administered a random survey involving 678 students between 18 and 30 years of age. Women exhibited a higher prevalence of acute tinnitus (≦1 year) than men did, possibly due to the reduced hearing protection that women possess (42). Kim et al. reported a higher adjusted OR of tinnitus for female study participants relative to their male counterparts (1), although other studies have reported a higher OR for men relative to women (39). This disagreement may be due to differences in working environments, ethnicity, study designs, and sample source diversity.

The main strengths of this study were its large sample size, rigorous diagnostic elements, and longitudinal retrospective cohort study design. Although the data were observational, the participants were selected randomly, and many sampling biases were prevented. However, this study had several limitations. First, because our data were from a secondary database, we had no data on some key variables, such as disease severity, occupation, environmental cues, laboratory findings, psychosocial status, and history of substance use or over-the-counter drug use. We could not establish how these unknown factors influenced the relationship between the two diseases in question. Second, tinnitus was diagnosed using the ICD-9 codes from the database, and its prevalence may have been underestimated because only patients seeking medical evaluation could be identified. However, this would most likely have resulted in an underestimation of the association between tinnitus and anxiety disorders. Third, although the data we obtained on tinnitus and anxiety disorder diagnoses were highly reliable, the diagnoses in NHI claims are primarily for administrative billing purposes and are thus not scientifically verified. Fourth, the results were based on a Taiwanese data set, potentially limiting their generalizability to the rest of the world. Further studies are required to address these limitations.

In conclusion, a significant increase was exhibited in the lifetime incidence of tinnitus in subjects with anxiety disorder. An age older than 60 years, female sex, and certain cardiovascular risk factors such as hypertension, and dyslipidemia constituted prominent risk factors for tinnitus in subjects with anxiety disorder. Although our study results suggest that anxiety is a possible cause of tinnitus, the exact mechanisms remain to be determined. More research in genetics and neurobiology along with large-scale epidemiological studies may provide additional clarity.

## Data Availability Statement

The Taiwan National Health Insurance Research Database (NHIRD) is managed by the National Health Research Institute (NHRI) and the NHIRD cannot be publicly available according to NHRI’s rules. Requests to access these datasets should be directed to NHRI.

## Ethics Statement

The Institutional Review Board of the Taipei Veterans General Hospital approved this study (VGHIRB No.: 2018-07-016 AC). Written consent from study participants was not obtained, because the NHI dataset comprises de-identified secondary data that can be used for research and the Institutional Review Board of Taipei Veterans General Hospital issued a formal written waiver for the requirement of consent.

## Author Contributions

Study conception and design: S-JH, C-CS, and T-HL. Acquisition of data: AY, C-CS, and S-JT. Analysis and interpretation of data: S-JH and C-CS. Draft manuscript: S-JH, C-CS, and T-HL. All authors contributed to the article and approved the submitted version.

## Funding

This work was supported by grant V108C-038 from the Taipei Veterans General Hospital and grant MOST 108-2314-B-367 -001 from the Ministry of Science and Technology. The funders had no role in the study design or procedures; in the collection, management, analysis, or interpretation of the data; in the preparation, review, or approval of the manuscript; or in the decision to submit the manuscript for publication.

## Conflict of Interest

The authors declare that the research was conducted in the absence of any commercial or financial relationships that could be construed as a potential conflict of interest.

## Abbreviations

CI, confidence Interval; DCN, dorsal cochlear nucleus; HPA, hypothalamic–pituitary–adrenal axis; HR, hazard ratio; ICD-9-CM, International Classification of Diseases, ninth revision, Clinical Modification; IQR, interquartile range; LHID 2005, Longitudinal Health Insurance Database 2005; LC, locus coeruleus; NHI, National Health Insurance; NHIR, National Health Research Institutes; NHIRD, NHI research database; NT$, new Taiwan dollars; OR, odds ratio; RR, rate ratio.
