# Supplementary material for: Tinnitus Among Patients With Anxiety Disorder: A Nationwide Longitudinal Study
Source: Front Psychiatry. 2020 Jun 25;11:606. doi: 10.3389/fpsyt.2020.00606 (PMC7329992; doi:10.3389/fpsyt.2020.00606)
Supplement: Supplementary file 1 [file Table_1.docx]

**Supplementary table 1 Risk Factors for Tinnitus in Patients with and without Anxiety Disorder (Anxiety disorder was diagnosed by psychiatrists or non-psychiatrists)**

| Predictive variables | | Univariable analysis | | | Multivariable analysis | | |
| --- | --- | --- | --- | --- | --- | --- | --- |
|  |  | HR (95% CI) | *P* value | | HR (95% CI) | *P* value | |
| Anxiety | | 1.86 (1.78-1.94) | <.001 | 1.80 (1.72-1.88) | | <.001* |  |
| Age (<60 = 0, ≥60 = 1) | | 0.78 (0.47-1.28) | .325 |  | |  |  |
| Sex (Female = 1, Male = 0) | | 0.99 (0.94-1.05) | .730 |  | |  |  |
| Comorbidities | |  |  |  | |  |  |
|  | Hypertension | 1.26 (1.18-1.35 | <.001 | 0.99 (0.91-1.07) | | .786 |  |
|  | Diabetes mellitus | 1.27 (1.17-1.37) | <.001 | 1.10 (0.99-1.20) | | .054 |  |
|  | Dyslipidemia | 1.52 (1.42-1.64) | <.001 | 1.28 (1.18-1.40) | | <.001* |  |
|  | Coronary artery disease | 1.17 (0.89-1.54) | .266 | |  |  | |
|  | Congestive heart failure | 1.25(1.07-1.46) | .005 | | 1.08 (0.90-1.29) | .431 | |
|  | Chronic lung disease | 1.29 (1.19-1.39) | <.001 | | 1.10 (1.01-1.20) | .037* | |
|  | Malignant neoplasms | 1.03 (0.82-1.27) | .824 | |  |  | |
|  | Head injury | 1.31 (1.22-1.42) | <.001 | | 1.16 (1.07-1.26) | .001* | |
|  | Cerebrovascular disease | 1.32 (1.18-1.49) | <.001 | | 1.13 (0.98-1.29) | .084 | |
| Degree of urbanization | |  |  | |  |  | |
|  | Urban | Reference |  | | Reference |  | |
|  | Suburban | 1.09 (1.03-1.16) | .005 | | 1.03 (0.96-1.10) | .378 | |
|  | Rural | 1.08 (0.99-1.19) | .095 | | 0.94 (0.85-1.04) | .244 | |
| Income group | |  |  | |  |  | |
|  | Low income | Reference |  | | Reference |  | |
|  | Medium income | 1.15 (1.08-1.22) | <.001 | | 1.12 (1.05-1.20) | .001* | |
|  | High income | 1.12 (1.02-1.24) | .024 | | 1.10 (0.99-1.23) | .077 | |

HR: hazard ratio; CI: confidence interval;

*statistical significance


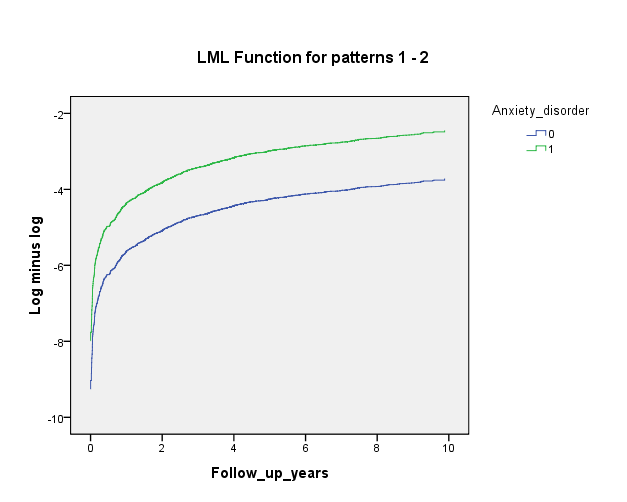


**Supplementary figure 1**

The log-minus-log survival plot, showing a constant increase in the cumulative hazard rate during the follow-up period.
